# SMART Pharmacist—The Impact of Education on Improving Pharmacists’ Participation in Monitoring the Safety of Medicine Use in Montenegro

**DOI:** 10.3390/pharmacy13020057

**Published:** 2025-04-17

**Authors:** Snežana Mugoša, Arijana Meštrović, Veselinka Vukićević, Milanka Žugić, Michael J. Rouse

**Affiliations:** 1 Institute for Medicines and Medical Devices of Montenegro (CInMED), 81000 Podgorica, Montenegro; snezana.mugosa@cinmed.me (S.M.); veselinka.vukicevic@cinmed.me (V.V.); 2 Faculty of Medicine, University of Montenegro, 81000 Podgorica, Montenegro; 3 Pharma Expert, 10040 Zagreb, Croatia; 4 Pharmaceutical Chamber of Montenegro, 81000 Podgorica, Montenegro; mzugic@gmail.com; 5 American Pharmacists Association, Washington, DC 20037, USA; mrouse@aphanet.org

**Keywords:** pharmacist education, patient care, adverse drug reaction, pharmacists’ intervention, patient safety

## Abstract

Pharmaceutical care as a concept was introduced in Montenegro during the last 10 years. The Pharmaceutical Chamber of Montenegro (PCM) and the Institute for Medicines and Medical Devices (CInMED) conducted SMART Pharmacist Program educational activities for pharmacists as a project to improve their impact on monitoring drug safety. In the period from September 2023 to May 2024, a total of 78 pharmacists participated in the project, of which 53 (68%) submitted valid reports of suspected adverse drug reactions (ADRs). During the project, a total of 302 valid reports were submitted, and the pharmacists’ share in total reporting increased to 74% in 2023 compared to less than 10% in the previous 5 years. The results of this research will be used to make recommendations for further improvement of the pharmacovigilance system, as well as to create plans for continuing education (CE) of other health workers in the area of rational and safe use of medicines.

## 1. Introduction

The World Health Organization (WHO) defines pharmacovigilance as a scientific discipline that includes activities related to the detection, collection, assessment, prevention of, and response to adverse effects of drugs, as well as other problems related to their use [1]. WHO defines adverse drug reactions (ADRs) as noxious and unintended responses to a medicinal product that occur at doses normally used for prophylaxis, diagnosis, or therapy of disease, or for the modification of physiological function [2]. According to the regulations of the European Union (EU) and the Law on Medicines (“Official Gazette of the Republic of Montenegro” No. 080/20), an adverse effect of a drug is considered to be any harmful and unintentionally caused effect. According to the Article 179 of the Law on Medicines, in Montenegro, all healthcare professionals (doctors, nurses, pharmacists) are obliged to report any suspected ADR—particularly serious and unexpected events—to the Institute for Medicines and Medical Devices of Montenegro (CInMED) [3]. A serious ADR should be reported within 30 days, while a fatal ADR should be reported as soon as possible. The obligation of CInMED, as a regulatory body, is to organize and improve the pharmacovigilance system. Additionally, CInMED aims to motivate health workers and patients to participate more actively in the system to recognize potential problems related to the use of drugs as quickly as possible and to respond in a timely manner.

In the period from September 2023 to May 2024, CInMED, in cooperation with the Pharmaceutical Chamber of Montenegro (PCM) and the Pharma Expert agency, implemented a SMART Pharmacist Program education and implementation project to develop the competence and motivation of pharmacists to recognize and report suspected ADRs, thereby becoming active participants in monitoring the safe use of drugs. The SMART Pharmacist Program was chosen for this intervention due to its proven effect on pharmacists’ behavior when implemented in Serbia and Turkey [4,5].

The SMART Pharmacist Program, first introduced in Turkey [5], is based on the Continuing Professional Development (CPD) approach to lifelong learning, with a particular emphasis on the application of learning in practice. The Accreditation Council for Pharmacy Education (ACPE, USA) defines CPD as “a self-directed, ongoing, systematic and outcomes-focused approach to lifelong learning that is applied into practice. CPD involves the process of active participation in formal and informal learning activities that assist individuals in developing and maintaining continuing competence, enhancing their professional practice, and supporting achievement of their career goals” [6]. The CPD Cycle (graphic representation) adopted by ACPE is used in the SMART Pharmacists Program. It depicts two joined cycles that portray the sequential elements of learning and application of learning [7].

In a SMART Pharmacist Program educational activity, the first component is a mandatory and in-depth introduction to the CPD approach to learning. The second component is the clinically focused element of the educational activity. After the completion of these two educational components, the participants are required to demonstrate the application of their learning in practice, typically through some form of patient interaction and collection of patient outcomes data or other measurable impact. The data are collected by the organization arranging the program and, in this way, participants are held accountable to demonstrate application and impact of learning. In addition, the program can be conducted as a study and the results published to provide evidence of the effectiveness of the educational strategy [4,5]. In contrast with traditional continuing education activities, which typically involve a single educational activity, the SMART Pharmacist Program approach involves multiple educational elements and data collection over a period of several months (usually 4–6).

The SMART Pharmacist Program was initiated in Montenegro by PCM through an asthma project in 2016–2019 and a post-COVID project in 2022. These projects emphasized the public health roles that pharmacists can have versus “clinical inertia”, providing evidence-based methods for learning and increasing competencies of pharmacists in the community [8]. This was additionally important to initiate, as the concept of pharmaceutical care in Montenegro was only regulated in 2015. It was an extensive task because “pharmaceutical health care” was not yet defined in the regulation of countries in the South and Eastern European (SEE) region. The PCM launched an initiative to advocate for the Ministry of Health to enact a law on pharmacy, which would describe pharmacy activity, pharmaceutical health care, and public health. A wide range of stakeholders were engaged, including experts from the International Pharmaceutical Federation (FIP) and WHO. As a result, for the first time, the law defined the term “pharmaceutical health care” in the description of activities performed by a pharmacist, thus paving the solid foundation for implementation of pharmaceutical services in the future [9].

In Article 20 of the Pharmacy Law in Montenegro, the term “pharmaceutical health care” was introduced, with the following aim: “In order to achieve better pharmacotherapeutic effects and implement rational consumption of drugs and medical devices and preserve health and prevent disease, in connection with the dispensing, i.e., proper application and storage of drugs and medical devices, the pharmacy can also implement pharmaceutical healthcare services” [9]. Some of those services include improving pharmacotherapeutic measures and procedures in the rational application of drugs and medical devices and providing information to the general and professional public about medication use; reporting ADRs and events involving medicines and medical devices, that is, reporting falsified medicines and pharmaceutical substances, in accordance with the law; as well as cooperation with other health care institutions and health care workers regarding the use of drugs and medical devices [9].

Pharma Expert, an educational agency and a Licensed Partner of the American Pharmacists Association (APhA), provided APhA’s *Delivering Medication Therapy Management Services Certificate Training Program* (CTP), presenting a systematic approach for developing, implementing, delivering, and sustaining Medication Therapy Management (MTM) services as the clinical part of the SMART Pharmacist Program. It includes guidance for implementing MTM services in pharmacy practice, a review of the essential skills and knowledge needed for performing MTM successfully, and an organized process for identifying medication-related problems [10]. The purpose of the CTP is to prepare pharmacists to improve medication use through the delivery of MTM services in a variety of practice settings.

The aim of the project was to determine the impact of the implementation of the SMART Pharmacist Program, including education and implementation of the MTM process, on the results of spontaneous reporting of ADRs in Montenegro. One of the most important indicators for this was to determine any increase in the ADRs that were recognized and reported by the pharmacists who participated in the project. The program was organized in three educational modules from September 2023 to May 2024 with required elements: introduction of the CPD model, introduction of the APhA MTM certificate program, and finally, presentation of collected patient cases, including submissions of ADR reports.

## 2. Materials and Methods

The call for education for the MTM service was addressed to all pharmacists who perform pharmaceutical activities in community pharmacies in Montenegro. Inclusion criteria were their membership in the Pharmaceutical Chamber of Montenegro with a valid work license and daily contact with the patients in their respective pharmacies. When the call was sent out, most participants in the SMART project were delegated by the management of the health institutions where they were employed. In all 55 pharmacies owned by the state (publicly owned), one pharmacist was delegated. The other 23 participants were voluntarily registered from privately owned pharmacies.

For the application of learning and to demonstrate the implementation of the patient care process, participants in the SMART Pharmacist Program were encouraged to report ADRs during the MTM process in practice. As the MTM procedure includes questions about patient safety, there is a certain point in the process when pharmacists need to collect patients’ feedback about possible ADRs. To evaluate the outcomes and impact of their learning, participants were asked to collect and report ADRs identified in the process of care, as a clear indicator of implementation of their learning.

Each ADR report was evaluated in the CInMED Pharmacovigilance Department. Reports that contained all necessary information about the patient, the suspected drug, the ADR and the reporter were considered as valid. One report was related to a medical device and one more to a dietary supplement. These reports were not considered but were forwarded to the appropriate department/institution. Additionally, two reports were related to suspected off-label use of the drug. These reports were also forwarded to the responsible department and archived but not evaluated as no ADR was reported. Additional information from reporters was required by CInMED pharmacovigilance staff when clarification was necessary to confirm report validity. This process increased participants’ awareness of the importance of submitting all available information in their case reports. All valid reports were assessed by experienced pharmacovigilance assessors based on the seriousness of the ADR reported. Feedback containing evaluation of the report and explanation of the purpose of ADR reporting was sent to the reporters by email. General feedback was also given by CInMED in the form of a presentation at the final educational meeting, when the main results and conclusions were discussed.

Data on ADRs reported to CInMED in the period from 2010 to 2023 were analyzed, as well as publicly available data on ADRs that were reported to regional agencies in that period, based on the type of reporter, the classification of the reported medicines, and the classification of ADRs due to severity. Medicines are listed according to the Anatomical Therapeutic Chemical (ATC) Classification, while ADRs are presented according to the Medical Dictionary for Regulatory Activities (MedDRA).

A retrospective analysis of reports of suspected ADRs submitted by project participants during the duration of the project was performed. The percentage share of reports submitted by pharmacists in the year of project implementation compared to previous years was also analyzed. The results are presented in textual, tabular, and graphical form, and descriptive statistics were used.

## 3. Results

A total of 78 pharmacists participated in the project, of which 53 (68%) submitted valid reports of suspected ADRs. During the project, a total of 302 valid reports were submitted; on average, project participants submitted 4 (3.87) reports of suspected ADRs.

**Figure 1:**
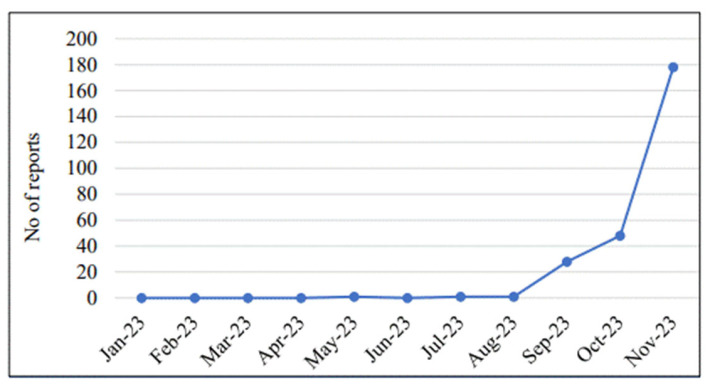
Number of ADR reports submitted by pharmacists in 2023, before and after the start of the SMART Pharmacist Program MTM project.

With regard to the timing of the reporting of ADRs during the project, it was noted that the largest number of reports was submitted in November 2023, while the implementation of the SMART Pharmacist Program MTM project began in September 2023 (Figure 1).

During the SMART Pharmacist Program project, the total share of reports submitted by pharmacists, both those employed in the private sector and pharmacists employed in state healthcare institutions, increased significantly. Pharmacists employed in privately owned pharmacies submitted a total of 133 (44%) reports, while pharmacists from state-owned pharmacies submitted 169 (56%) reports. Reports were submitted from several different cities. As expected, the largest number of reports was submitted from the capital city of Podgorica.

The largest number of ADR reports (206, or 68.2% of the total received) were submitted through the online process due to its simplicity and accessibility; access was through the CInMED portal (e reporting). A significant number of reports, 40 (13.2%), were submitted through the IS ZU Pharmacy of Montenegro “Montefarm”, which confirmed the importance of a direct IT connection with CInMED for pharmacists employed in the state sector. A total of 39 reports (12.9%) were submitted by email, while 17 reports (5.6%) were submitted by mail.

After validation, all reports were evaluated, included into the national database on ADRs, and forwarded to the global database of the WHO Programme for International Drug Safety Monitoring—VigiBase.

### 3.1. The Severity of the Adverse Drug Reactions

Out of a total of 302 reports of suspected ADRs, 35 reports (12%) met at least 1 of the severity criteria. The remaining 267 reports (88%) were for ARDs that were classified as not serious.

In 29 (9.6%) of the 302 reports, a clinically significant condition requiring immediate intervention was reported as a criterion for severity. Hospitalization or prolonged hospitalization was listed as the reason for severity in 8 reports (2.6%), where 1 report may meet more than 1 severity criterion. For example, one reported case may relate to multiple ADRs, some of which required immediate intervention and then hospitalization of the patient. Five reports (1.6%) were related to a condition that led to a life-threatening condition of the patient, and no fatal cases were reported. The coding of ADRs was performed using the *Medical Dictionary for Regulatory Activities (MedDRA)*.

There was no safety signal detected based on these reports. One report described a serious allergic reaction after tolperisone use in an off-label indication of spinal pain. This initiated a national database analysis and redistribution of the Direct Health Professional Communication (DHPC) in April 2024 as a reminder that tolperisone use should be restricted to the treatment of adults with post-stroke spasticity (stiffness) only. No other regulatory actions were taken.

The analysis of reports by the gender of the patient revealed that a higher number of reports of ADRs referred to women, which is also the case in other countries [11]. A total of 208 reports (69%) were related to female patients, of which 3 (1%) were related to the use of the drug during pregnancy. In 94 reports (31%), ADRs occurred in male patients.

The number of reported ADRs by patient age is shown in Table 1. The largest number of reports (134, 44.4%) were submitted in adult patients aged 45 to 64. Patients aged 18 to 44 years accounted for 79 reports (26.1%). A significant number of reports were submitted for elderly patients: a total of 50 reports were submitted for patients aged 65 to 74 years, and 30 reports for patients over 75 years of age.

Using International Nonproprietary Names (INNs), the most commonly suspected active substances were atorvastatin, diclofenac, ibuprofen, and metformin: 10 (3.3%) reports each. Most frequently, reports were related to generics, which are commonly used in Montenegro. There were no reports associated with the use of investigational medicinal products during clinical trials.

The analysis of reports in relation to Anatomical–Therapeutic–Chemical (ATC) Classification of a suspected medicinal product is shown in Table 2. Since 1 reported case may relate to the effects of 1 or more suspected drugs used, in a total of 302 reports submitted to CInMED within the project, 314 drugs were suspected of causing an ADR. According to the ATC classification of those drugs, the largest number of submitted reports was related to drugs belonging to Group C—Cardiovascular System, which is expected given that drugs for the treatment of cardiovascular diseases are the most commonly used drugs in Montenegro. A significant number of reports also relates to drugs from Group J—Anti-infective drugs for systemic use, which includes antibiotics and vaccines, which is also in line with the CInMED’s data on the consumption of these drugs.

### 3.2. The Most Common Reported Adverse Drug Effects

Since it is possible for a single patient to experience more than one effect of a particular medicine at the same time, the number of ADRs reported is significantly higher than the number of reports received. Reports submitted within the project contained a total of 641 ADRs. Bearing in mind that the total number of reports submitted was 302, each report described, on average, slightly over 2 (2.1) ADRs. Most of the reports were related to the expected ADRs. Out of 302 valid reports, only 35 (11.6%) contained at least one reaction which was not labeled in the respective Summary of Product Characteristics (SmPC). However, there was no safety signal detected based on these reports.

**Figure 2:**
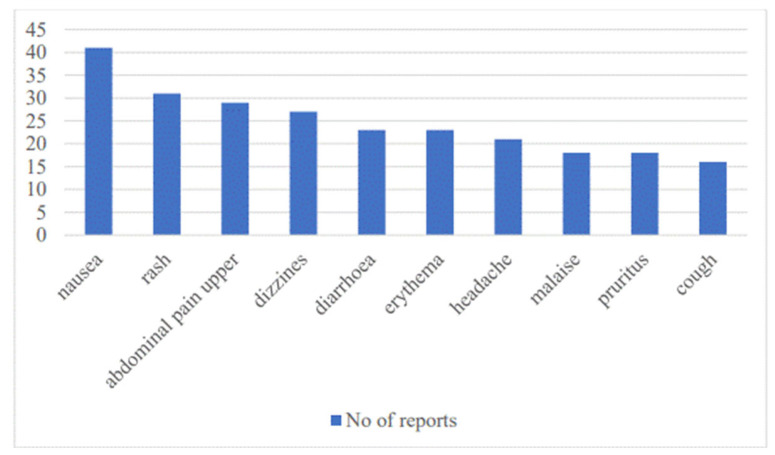
The most commonly reported ADRs in the SMART Pharmacist Program Project in Montenegro (MedDRA PT level).

Figure 2 is an overview of the most frequently reported ADRs during the project, presented according to the preferred term (Preferred Term, PT) given by the Medical Dictionary for Regulatory Activities (MedDRA).

**Figure 3:**
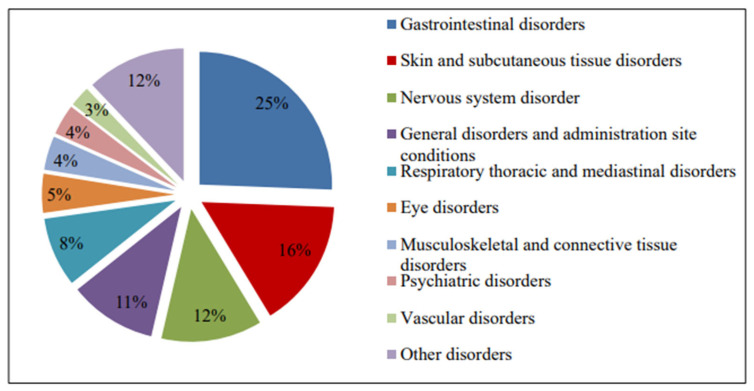
The ADRs reported according to MedDRA System Organ Class (SOC).

Figure 2 shows that the most commonly reported ADRs are nausea, rash, upper abdominal pain, dizziness and diarrhea. The Medical Dictionary for Regulatory Activities (MedDRA) was used to code ADRs. The ADRs reported on the basis of the MedDRA System Organ Class (SOC) in most cases belong to the following categories: gastrointestinal disorders (163, 25.4%), skin and subcutaneous tissue disorders (100, 15.6%), nervous system disorders (78, 12.2%), and general disorders and administration site conditions (68, 10.6%), as shown in Figure 3.

**Figure 4:**
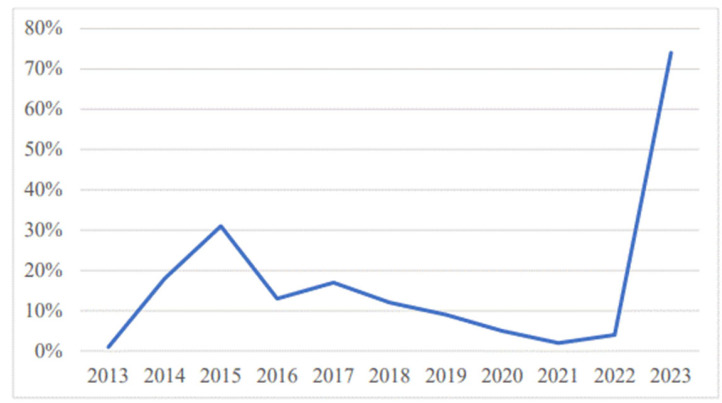
Pharmacists’ share in reporting ADRs in Montenegro from 2013 to 2023.

The share of reports submitted by pharmacists during the SMART Pharmacist Program project (2023) was 74%. According to the available annual reports of reported ADRs, the share of reports submitted by pharmacists in the total reporting in the previous ten-year period ranged from a minimum of 1% in 2013 to 31% in 2015. The increase in the number of reports submitted by pharmacists in 2015 would appear to be related to the educational workshops that were organized during this period. In 2023, the number of reports submitted by pharmacists was significantly higher, as shown in Figure 4.

## 4. Discussion

### The Impact of the Implementation of the SMART Pharmacist Program Project on the Share of Applications Submitted by Pharmacists in Previous Years

Participants were encouraged to advance pharmacovigilance procedures, and their ability and motivation to recognize and report ADRs improved, which was the goal of the project. As a result, the number of reports submitted by pharmacists increased significantly, as the share of reports submitted by pharmacists was 74% in 2023, compared with less than 10% in each of the previous 5 years. As discussed later, the authors believe that this increase is the direct result of the implementation of the SMART Pharmacist Program project, as shown by the fact that out of a total of 293 reports submitted by pharmacists during 2023, 284 (96.9%) were submitted by project participants. In the same year, according to publicly available annual reports of regulatory authorities, the number of reports submitted by pharmacists accounted for 13.5% of the total number of submitted reports in Serbia [12], and 15.5% of the total number of submitted reports in Croatia [13].

Adverse effects of drugs represent a great burden for patients and on health systems; they are the cause of 3.5% of hospitalizations in EU countries, and they are in 4th to 6th place according to the cause of death in the USA [14,15]. In addition, research shows that over 50% of ADRs are preventable, which further indicates the importance of their recognition and reporting. Although the spontaneous reporting of suspected ADRs by health workers and patients is the basis of the pharmacovigilance system, in Montenegro, as well as globally, a large number of manifested ADRs remain unreported.

Under-reporting is recognized as one of the key reasons for late recognition and untimely response to ADRs. Pharmacists, as the most accessible healthcare workers, are in an ideal position to influence the correct use of medicines and contribute to better treatment outcomes. The research conducted by the Netherlands Pharmacovigilance Centre Lareb included a total of 15,293 reports submitted to this center between 1995 and 2000. The results of this research showed that pharmacists submitted 40% of the total number of reports submitted in the specified period, and that the contribution of pharmacists to the national pharmacovigilance system is significant both in terms of the number and quality of the submitted information [16]. However, pharmacists’ engagement in the pharmacovigilance system is not equally represented in all countries. Research conducted in Great Britain, which is one of the countries with the most developed pharmacovigilance system, showed that pharmacists have some knowledge of the system of spontaneous reporting of ADRs, but that their education is still necessary to maintain and increase the number of reports they submit [17]. However, in Scandinavian countries, pharmacists still do not have the right to report ADRs, while in those countries where they have this right, pharmacists often do not use it. Reports submitted by pharmacists are valued more by those countries where pharmacists submit a larger number of applications compared to countries where the number of applications submitted by pharmacists is not significant [18]. In addition, pharmacists who have completed appropriate educational activities can influence greater involvement of patients in spontaneous reporting [19].

The analysis of the attitudes of pharmacists conducted by Hadi et al. in 2017 showed that pharmacists undoubtedly play a significant role in the detection, reporting, and prevention of ADRs. However, a lack of adequate and continuing education in this area can result in an ineffective pharmacovigilance system, which ultimately can threaten the safety of patients [20]. This research has provided insights into the impact of an intervention in the form of an educational project on the practice of reporting suspected ADRs as well as on the characteristics of recognized and reported ADRs.

The project also included a retrospective pharmacoepidemiologic study to compare rates of individual case safety reports (ICSRs) and characteristics among some South and Eastern European (SEE) countries, Croatia, Serbia, Montenegro, and Bosnia and Herzegovina (B&H), using the data from ICSRs received by the Agency for Medicines and Medical Devices in B&H in 2011–2016 [21]. In Croatia, the number of reported ADRs per one million inhabitants was the highest, while Bosnia and Herzegovina (B&H) had the lowest. Serbia and Montenegro reported similar numbers, falling between the two [22]. In Serbia, despite the community pharmacists’ positive attitude toward ADR reporting and their role in the process, they show limited knowledge regarding the issue and highly prevalent under-reporting of ADRs. Educational programs are necessary to increase ADR reporting [23].

In Montenegro, the Agency for Medicines and Medical Devices (CInMED) plays a vital role in advancing pharmacovigilance and education. The reporting system is being continuously improved, particularly focusing on online options. The online forms are well-structured, featuring many mandatory fields. Additionally, CInMED has developed various projects and collaborations to enhance the pharmacovigilance system.

There were significant differences in reporter characteristics, sources of reports, and the percentage of missing data in ICSRs, while the Anatomical Therapeutic Chemical (ATC) product classes, patient’s gender, and ADR System Organ Classes were similar. Despite the historical and geographic vicinity of those neighboring countries, there were significant differences in indicators of pharmacovigilance development [20].

Although 78 pharmacists participating in the SMART project gained valuable practical knowledge on ADR reporting, this is approximately 14% of all active pharmacists in Montenegro. Further education activities are needed to include all pharmacists and generally improve their participation in the pharmacovigilance system.

In 2016, a publication described the pharmacovigilance experience from Montenegro, identifying the potential of future developments of the ADR reporting system. As the effectiveness of each country’s pharmacovigilance system relies heavily on the involvement of healthcare professionals, to promote pharmacovigilance, CInMED undertook various initiatives that emphasized the significance of spontaneous reporting of ADRs and the training of healthcare professionals in this area. It was planned to organize workshops focused on pharmacovigilance, aimed at establishing a system for the continuous monitoring of medication safety, and this SMART Pharmacist Program project was part of this plan, as an intervention to increase ADR reporting in Montenegro [24]. Based on the research, a model for improving the pharmacovigilance system in Montenegro will be proposed to enable more active participation of pharmacists and strengthen their role in the health system. CInMED is continuously improving and designing new ways to report ADRs, to make the reporting process as simple as possible for healthcare professionals and patients and better monitor the safety of drug use in Montenegro.

There are recently published systematic reviews and meta-analyses on the effectiveness of strategies for enhancing adverse drug reaction reporting. Meta-analysis showed significant increases in overall ADR reporting through educating HCPs through face-to-face interactive workshops. Email or letter communications showed no significant effect. More studies are needed to confirm these findings before recommending widespread implementation in clinical practice [25].

One of the central principles and the primary goal of the SMART Pharmacist Program is that learning is applied in practice (*“Learn Today—Apply Tomorrow”*) and—as a result—educational activities have a measurable impact on patient care outcomes and/or improve the quality of services provided by the pharmacist [8]. In contrast to traditional approaches to continuing education (CE) that typically only measure *participation* in educational activities (CE hours, credits, or points), the SMART Pharmacist Program requires participants to demonstrate the *application of the learning* through the collection and analysis of relevant patient and/or service-related data [3,4,5].

## 5. Conclusions

Implementation of the SMART Pharmacist Program project as a strategy to educate and encourage pharmacists to spontaneously report ADRs as a component of a Medication Therapy Management (MTM) service led to a significant increase in the number of submitted reports and the percentage of reports submitted by pharmacists during the project. The project demonstrated the positive impact that can be achieved through an appropriately designed educational intervention. Recognizing that pharmacists are an unparalleled source of information on the safe use of drugs and pharmacists in community settings are the most accessible healthcare providers, it is clear that the success of a pharmacovigilance system depends to a large extent on their engagement. The results of this research will be used to make recommendations for further improvement of the pharmacovigilance system, as well as to create plans for continuing education of pharmacists and other health workers in the area of rational and safe use of medicines. In addition, based on the results of the research and lessons learned from the SMART Pharmacist Program, models for the education of other healthcare workers in Montenegro will be proposed to develop awareness of the importance of spontaneous reporting of ADRs.

## Figures and Tables

**Table 1:** Number of ADR reports by patient age.

AGE GROUP	*NUMBER OF REPORTS*
0–27 days	0 (0%)
28 days–23 months	2 (0.7%)
2–11 years	7 (2.3%)
12–17 years	0 (0%)
18–44 years	79 (26.1%)
45–64 years	134 (44.4%)
65–74 years	50 (16.5%)
≥75 years old	30 (9.9%)
*TOTAL NUMBER OF REPORTS*	302 (100%)

**Table 2:** Number of reports in relation to Anatomical–Therapeutic–Chemical (ATC) Classification of a Suspected Medicinal Product.

ATC	ATC Classification Core Group	Number of Reported Medicines
**A**	Alimentary tract and metabolism (medicines that act on diseases of the digestive system and metabolism)	**40**
**B**	Blood and hematopoietic organs (medicines used to treat blood and hematopoietic organs)	**22**
**C**	Cardiovascular system (medicines that act on the cardiovascular system)	**84**
**D**	Skin and subcutaneous tissue (medicines used to treat diseases of the skin and subcutaneous tissue)	**7**
**G**	Genitourinary system and sex hormones (medicines for the treatment of the genitourinary system and sex hormones)	**13**
**H**	Hormonal preparations for systemic administration, excluding sex hormones and insulin	**3**
**J**	Anti-infective drugs for systemic use	**45**
**L**	Antineoplastics and immunomodulators	**9**
**M**	Musculoskeletal system (medicines for diseases of the musculoskeletal system)	**33**
**N**	Nervous system (medicines that act on the nervous system)	**30**
**P**	Antiparasitic products, insecticides and insect repellents (medicines for the treatment of infections caused by parasites)	**4**
**R**	Respiratory system (medicines to treat diseases of the respiratory system)	**18**
**W**	Sensory organs (medicines that affect the eye and ear)	**6**
**V**	Miscellaneous	**0**
	**TOTAL NUMBER OF MEDICINES**	**314**

## Data Availability

Data supporting reported results can be found in the archive of CInMED, including links to publicly archived datasets analyzed or generated during the study.
